# Synchronous Carotid Bifurcation Endarterectomy and Retrograde Kissing Stenting of the Innominate and Left Common Carotid Artery in a Patient with a Bovine Aortic Arch

**DOI:** 10.1155/2017/4239829

**Published:** 2017-04-11

**Authors:** Alessandro Robaldo, Guido Carignano, Alberto Balderi, Claudio Novali

**Affiliations:** ^1^Vascular Surgery Unit, Santa Croce and Carle Hospital, Via Michele Coppino 26, 12100 Cuneo, Italy; ^2^Department of Radiology, Santa Croce and Carle Hospital, Via Michele Coppino 26, 12100 Cuneo, Italy

## Abstract

Management of the symptomatic multiple stenosis of supra-aortic vessels (MSSVs) in a “bovine” aortic arch (BAA) configuration is infrequently reported. The optimal treatment choice remains debatable. A successful hybrid treatment for a proximal critical stenosis of the innominate and left common carotid artery was performed in a high-risk patient with a tandem symptomatic lesion in the right carotid bifurcation and a concentric vulnerable plaque in the bovine trunk. This case supports the feasibility, safety, and efficacy of a combined carotid bifurcation endarterectomy and retrograde kissing stenting of common carotid arteries with cerebral protection after evaluation of radiological, anatomical, and clinical parameters.

## 1. Introduction

Multiple stenoses of supra-aortic vessels (MSSVs) are relatively uncommon. Most of these lesions are atherosclerotic and occur at the vessel origin. The association of this condition with a “bovine aortic arch” (BAA) is infrequent. However, diagnosis by noninvasive means is becoming increasingly more common and the preventive therapy is recommended because MSSVs may cause ischemic stroke, as may carotid bifurcation disease, with an incidence of 1-2% [1]. The conventional surgical repair via transthoracic approach was initially recommended [2]. However, due to the high mortality and complication rates, less-invasive extrathoracic approaches, including transfemoral percutaneous transluminal angioplasty (t-PTA) and stent-supported t-PTA, are described to obtain safe access and treatment for MSSV. In case that these approach results are technically difficult, alternative vascular accesses, as retrograde direct carotid approach, are to be considered [3]. We describe a successful hybrid treatment for a proximal critical lesion of the innominate and left common carotid artery in a high-risk patient with a tandem symptomatic right internal carotid stenosis and a concentric vulnerable plaque in the bicarotid common trunk. The patient consented to the publication of this report.

## 2. Case Report

**Figure 1:**
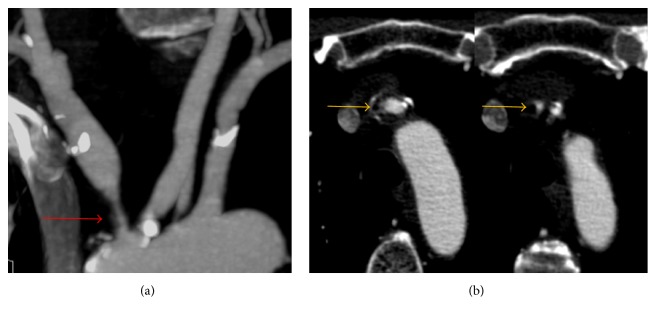
(a) A sagittal plane of the* CT angiography* shows critical stenosis of innominate and left carotid artery emerging from a bovine arch (red arrow). (b) In the axial plane, a vulnerable plaque includes the common trunk and the origin of the innominate and left common carotid artery (yellow arrows).

**Figure 2:**
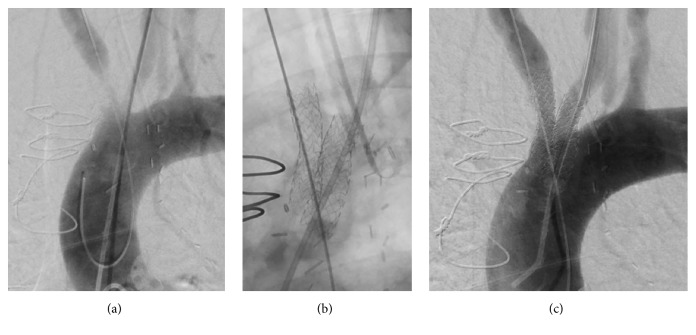
(a) Baseline angiogram of severe stenosis of the innominate and left common carotid arteries. (b) Omnilink stents across both vessels advanced over both wires in the common carotid arteries. (c) Final angiogram after stenting with antegrade flow in both vessels.

**Figure 3:**
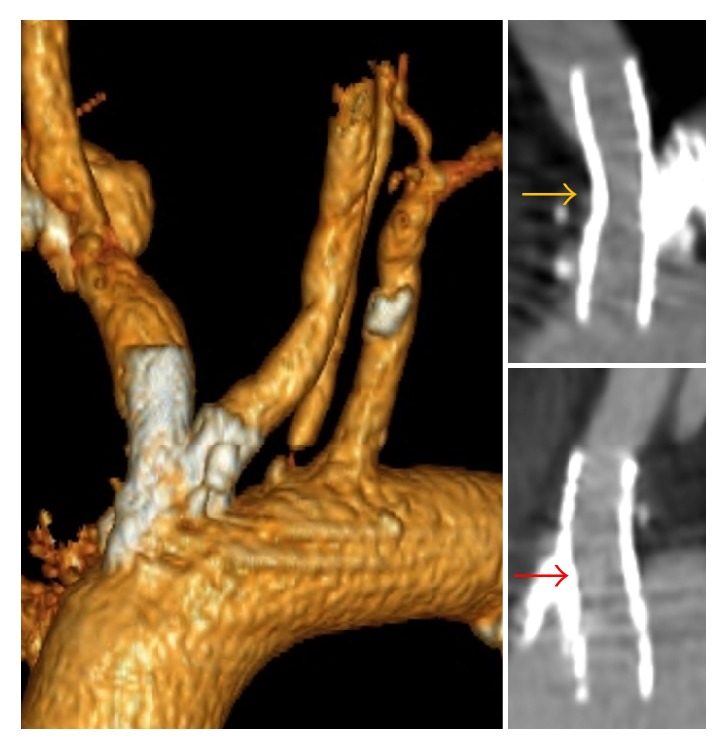
3-month CT angiogram demonstrates excellent dilatation of the innominate (yellow arrow) and left common carotid artery (red arrow) in the supra-aortic portion.

A 60-year-old Caucasian female with a history of heart failure, diabetes, dyslipidemia, and previous coronary artery bypass grafting was referred to our department for evaluation after an episode of confusion associated with dysphasia and amaurosis fugax of the right eye. Magnetic resonance of the brain 24 hours after admission did not reveal an acute lesion. A carotid duplex scan revealed 80% (NASCET) hemodynamic stenosis of the right internal carotid artery (ICA) and a poststenotic flow in both common carotid arteries. A contrast-enhanced multidetector computed tomography angiography (MDCTA) confirmed the lesion in the right carotid bifurcation and showed a proximal critical stenosis of the innominate and left carotid artery emerging from a BAA with a concentric vulnerable plaque of the bovine common trunk (Figure 1). Additional findings included stenosis of the nondominant left vertebral artery emerging directly from the aortic arch. Preoperative cardiac evaluation was assessed and the patient was considered to have a “grade III preoperative risk” according to the American Society of Anesthesiologists (ASA) classification. After a multidisciplinary consultation, which stated that all the carotid lesions were potentially congruent with the neurological symptoms, a hybrid treatment was chosen: (1) a kissing stenting of common carotid arteries via a bilateral direct carotid access and (2) a right internal carotid endarterectomy (CEA). Written informed consent was obtained from the patient who refused the regional anesthesia. The procedure was performed in a dedicated hybrid operating room with the use of high-resolution imaging (Artis Zeego; Siemens, Enlargen, Germany). Detection of cerebral ischemia was assessed with a continuous transcranial cerebral oximetry monitoring system (INVOS™ 5100C, Somanetics Corporation, Michigan, USA). A 300 mg clopidogrel preloading was administered. Under general anesthesia, both carotid bifurcations were exposed and internal carotid artery was isolated with vessel loops. An activated clotting time was maintained between 200 and 250 seconds after a bolus intravenous injection of 5000 UI of heparin. On both sides, an 18-gauge needle was introduced retrogradely into the proximal healthy segment of the common carotid arteries and 25 cm long 6 Fr sheaths were placed carefully at the distal portion of the stenosis under fluoroscopic control. A preliminary angiography from the sheath side arm showed no extracranial-intracranial anastomosis between the internal and the external carotid artery. The preoperative artery measurements were confirmed and the reference points were marked. After blood pressure was pharmacologically increased, a conventional simultaneous 8 × 29 mm balloon expandable kissing stenting (Omnilink Elite®, Abbott Vascular, Inc.) was performed at the origin of the right and left common carotid artery during a transient bilateral ICA looping to flush debris in the external carotid artery (Figure 2). The stents were brought inside the sheaths which were pushed through the lesions over an exchange length 0.035′′ Amplatz super-stiff guidewire. The clamping time was of 180 seconds and the INVOS system did not detect significant changes in the cerebral tissue saturation. After the final angiography revealed excellent dilatation of both stents and absence of residual disease, a flush was performed from the side port of the left sheath which was finally removed with a Z-stitch placed around the entrance of the sheath to facilitate rapid hemostasis without need of further carotid clamping. Subsequently, on the right side, a standard carotid bifurcation endarterectomy with Dacron patch angioplasty was achieved with the use of the Pruitt-Inahara shunt after the removal of the plaque. Immediate technical success was achieved without complications and the postoperative course was uneventful. The patient was discharged after 48 hours with a 3-month dual antiplatelet therapy. Follow-up at 3 months after device placement, including neurological observation, and MDCTA, demonstrated no complications, with patency of both proximal and distal treated lesions (Figure 3).

## 3. Discussion

The MSSVs represent a relatively rare entity with an incidence of less than 5% [4]. The optimal treatment choice remains complex and debatable, in particular when these lesions are associated with the most common anatomical variant of the aortic arch branching, the so-called “bovine arch.” The more usual subtype is described as the common origin of the brachiocephalic and left common carotid arteries and can occur in as many as 20% of patients [5, 6]. Although standard open surgical repair via transthoracic approach initially showed good early and long-term results [3], at present, due to the high rate of complications and mortality, this treatment has generally reserved for extensive multivessel involvement or after failed endovascular procedures in low-risk patients. T-PTA and stent-supported t-PTA in symptomatic atherosclerotic disease of the supra-aortic vessels have evolved as less-invasive safe access and treatment for MSSVs since the 1990s [7]. Nevertheless, even those techniques are not complication-free and are technically difficult in 1-2% due to several anatomical predicting factors. The severity of the stenosis, the elongation of the vessel, and aortic arch anomalies such as BAA can lead to increased neurological complications after CAS [8]. Indeed, the introduction of a large-caliber guiding catheter into the narrow origin of common carotid arteries carries the risk of cerebral embolization before activation of the protection device or acute dissection. In addition, the distance between the lesion and the aortic arch can be too short to support the introducer sheath with a higher risk of stent migration into aorta. Under these conditions, the use of alternative vascular accesses, as the retrograde direct carotid approach, has shown promising results [9]. In the present case, the retrograde stenting with cerebral protection by ICA clamping was considered the best technical solution due to the presence of both an eccentric vulnerable plaque in the short common trunk and a high-grade stenosis at the origin of the carotid arteries. Compared with other reports in which only isolated lesions were treated, the common origin of the stenotic carotid arteries required a simultaneous transient clamping of both ICAs to avoid distal embolization and flushing of the debris to the ECA territory during the guidewire manoeuvre and stent deployment. To our knowledge, only in one case, a similar bilateral carotid stenting was performed but without surgical access and any cerebral protection modalities [10]. The application of this technique is based on the fact that the cerebral parenchyma may tolerate, under general anesthesia, sufferance due to carotid clamping without neurological deficits for at least 7 minutes, as previously reported in some studies regarding the threshold ischemia and the delayed insertion of the shunt during carotid endarterectomy, even with contralateral carotid occlusion [11]. Even though some authors suggest to clamp the common carotid before the insertion of the sheath, we believed it is not feasible in a case of double ICA clamping because of the required longer time of brain ischemia. In case of an anomalous ECA-ICA anastomosis, the ECA should be clamped proximal to the ostium of the occipital artery and debris should be flushed into the superior thyroid artery. As far as the best type of anesthesia to use is concerned, in our case, we were forced to choose general anesthesia after consultation with the patient. However, it is reported that regional anesthesia with cervical block offers the advantage of a shorter ICU and hospital stay in addition to a continuous neurological monitoring [12]. Percutaneous carotid access is considered possible when CEA is not planned, but a surgical access seems to be safer. A protection against embolism and a quick hemostasis is achieved without exposing the patients to the risk of neck hematoma, pseudoaneurysm, tracheal compression, infection, and arteriovenous fistula in case of manual compression or vascular closure devices failure. Recent data report that the stroke and death rate for CEA with proximal endovascular intervention is higher than that of isolated CEA [13]. Hence, larger studies would be necessary to determine the clinical role of the technique, in particular in the asymptomatic patients. From a technical point of view, preoperative otorhinolaryngoiatric visit and careful carotid dissection are recommended to avoid the eventual life-threatening condition of bilateral vocal cord paralysis.

## 4. Conclusion

This paper suggests that the retrograde carotid kissing stenting with surgical cerebral protection could be a safe and effective solution to treat proximal MSSVs, especially in case that the use of the endovascular transfemoral approach is difficult because of the presence of potential embolic lesions and bovine arch.
